# P2Y12 receptor mediates microglial activation via RhoA/ROCK pathway in the trigeminal nucleus caudalis in a mouse model of chronic migraine

**DOI:** 10.1186/s12974-019-1603-4

**Published:** 2019-11-13

**Authors:** Feng Jing, Yixin Zhang, Ting Long, Wei He, Guangcheng Qin, Dunke Zhang, Lixue Chen, Jiying Zhou

**Affiliations:** 1 Department of Neurology, The First Affiliated Hospital of Chongqing Medical University, 1st Youyi Road, Yuzhong District, Chongqing, 400016 China; 2 Laboratory Research Center, The First Affiliated Hospital of Chongqing Medical University, Chongqing, China

**Keywords:** P2Y12 receptor, Microglia, Chronic migraine, Trigeminal nucleus caudalis, Hyperalgesia, Nitroglycerin

## Abstract

**Background:**

Microglial activation contributes to the development of chronic migraine (CM). The P2Y12 receptor (P2Y12R), a metabolic purinoceptor that is expressed on microglia in the central nervous system (CNS), has been indicated to play a critical role in the pathogenesis of chronic pain. However, whether it contributes to the mechanism of CM remains unknown. Thus, the present study investigated the precise details of microglial P2Y12R involvement in CM.

**Methods:**

Mice subjected to recurrent nitroglycerin (NTG) treatment were used as the CM model. Hyperalgesia were assessed by mechanical withdrawal threshold to electronic von Frey and thermal withdrawal latency to radiant heat. Western blot and immunohistochemical analyses were employed to detect the expression of P2Y12R, Iba-1, RhoA, and ROCK2 in the trigeminal nucleus caudalis (TNC). To confirm the role of P2Y12R and RhoA/ROCK in CM, we systemically administered P2Y12R antagonists (MRS2395 and clopidogrel) and a ROCK2 inhibitor (fasudil) and investigated their effects on microglial activation, c-fos, and calcitonin gene-related peptide (CGRP) expression in the TNC. To further confirm the effect of P2Y12R on microglial activation, we preincubated lipopolysaccharide (LPS)-treated BV-2 microglia with MRS2395 and clopidogrel. ELISA was used to evaluate the levels of inflammatory cytokines.

**Results:**

The protein levels of P2Y12R, GTP-RhoA, ROCK2, CGRP, c-fos, and inducible nitric oxide synthase (iNOS) in the TNC were increased after recurrent NTG injection. A double labeling study showed that P2Y12R was restricted to microglia in the TNC. MRS2395 and clopidogrel attenuated the development of tactile allodynia and suppressed the expression of CGRP, c-fos, and GTP-RhoA/ROCK2 in the TNC. Furthermore, fasudil also prevented hyperalgesia and suppressed the expression of CGRP in the TNC. In addition, inhibiting P2Y12R and ROCK2 activities suppressed NTG-induced microglial morphological changes (process retraction) and iNOS production in the TNC. In vitro, a double labeling study showed that P2Y12R was colocalized with BV-2 cells, and the levels of iNOS, IL-1β, and TNF-α in LPS-stimulated BV-2 microglia were reduced by P2Y12R inhibitors.

**Conclusions:**

These data demonstrate that microglial P2Y12R in the TNC plays a critical role in the pathogenesis of CM by regulating microglial activation in the TNC via RhoA/ROCK pathway.

## Background

Migraine is one of the most common disorders, and converts to a chronic condition in 2–3% of patients [1], although episodic migraine is more common, chronic migraine (CM) is more disabling, and patients experience a headache attack at least 15 days per month [2]. However, the current effective treatments for CM are limited [3], and our understanding of its mechanism is poor. One of the leading theories suggest that central sensitization, which manifests as an increase in the excitability of central neurons in the trigeminal nucleus caudalis (TNC), is critical in the pathogenesis of CM [4, 5] and that glia in the central nervous system (CNS) are also involved in this process [6–8]. Our previous studies showed that microglia were activated in the TNC in a CM model [9, 10]. Activated microglia may mediate nociceptive signals through chemotaxis, releasing pro-inflammatory cytokines and neurotrophins, and through microglial-neuronal interactions [11–13]. Therefore, microglia may be a new therapeutic target for treating CM.

Extensive evidences have shown that microglial purinoceptors such as P2X4R and P2X7R contribute to inflammatory pain, neuropathic pain, and migraine [9, 10, 14–16]. P2Y12R, another metabolic purinoceptor that is restricted to microglia in the CNS [17], has also been implicated in neuropathic, inflammatory, and cancer pain [18–20] and may regulate spinal microglial activation [21, 22]. However, whether P2Y12R is involved in the development of CM has not yet been studied. Several clinical studies have shown that clopidogrel, a common antithrombotic drug that inhibits the activity of P2Y12R, may prevent migraine attacks [23–25]. Thus, it is reasonable to predict that P2Y12R is involved in the pathogenesis of CM. In the present study, we first investigated the role of P2Y12R in CM using a chronic nitroglycerin (NTG) administration-induced CM animal model.

Previous studies showed that P2Y12R may participate in neuropathic pain by regulating the activation of RhoA and Rho-associated coiled-coil-containing protein kinase (ROCK) [18, 21]. RhoA, a small molecular G-protein, circulates between active (GTP-bound) and inactive (GDP-bound) forms. GTP-RhoA is involved in regulating cellular functions via interaction with downstream ROCK [26]. ROCK is a serine–threonine protein kinase containing two isomers, ROCK1 and ROCK2; ROCK2 is mainly expressed in brain tissues [27]. RhoA/ROCK participate in various physiological processes, such as actin cytoskeleton reorganization, cell growth control, chemotaxis, and morphological changes, and these proteins are also involved in inflammation by regulating the activation of p38 MAPK [18, 26]. However, whether they contribute to the mechanism of CM has not been studied.

Based on these data, we hypothesized that P2Y12R mediates the activation of microglia in the TNC via the RhoA/ROCK pathway and contributes to the mechanisms of CM. In this study, we confirmed the role of microglial P2Y12R and RhoA/ROCK2 in the TNC in a recurrent NTG application-induced CM model, using antagonists of these proteins. We also demonstrated the effect of P2Y12R on microglial morphological changes and inflammation in the TNC in CM model and in lipopolysaccharide (LPS)-stimulated BV-2 microglia. The results suggest that P2Y12R may be a new target for the treatment of CM.

## Methods

### Animals

Male C57BL/6 mice (20 g–30 g) were used in this experiment and were provided by the Experimental Animal Center of Chongqing Medical University (Chongqing, China). Animals were housed under controlled conditions with a 12 h/12 h light/dark cycle, temperature of 23 ± 2 °C, humidity of 50 ± 10%, and with food available ad libitum. All animal experimental procedures were approved by the local animal care committee and were in accordance with the animal care guidelines of the National Institutes of Health. We made every effort to reduce the number of animals used in the experiments and minimize animal suffering. Animals were randomly assigned to experimental groups.

### Drug administration

Nitroglycerin (NTG) (Beijing Reagent, China) was prepared from a stock solution of 5.0 mg/ml dissolved in water, 30% propylene glycol, and 30% alcohol. For the injections, NTG was freshly diluted to a dose of 1 mg/ml with saline, containing 6% propylene glycol and 6% alcohol. The diluted NTG was administered intraperitoneally (i.p.) at a dose of 10 mg/kg every other day for 9 days (i.e., days 1, 3, 5, 7, and 9) [28]. An equivalent volume of saline, 6% propylene glycol, and 6% alcohol was used as the vehicle.

To detect the precise role of P2Y12R in CM, animals were injected i.p. with the selective P2Y12 antagonists MRS2395 (1.5 mg/kg; Sigma-Aldrich, MO, USA) and clopidogrel (15, 45, and 100 mg/kg; Selleck, TX, USA) five times, every other day prior to NTG injections. MRS2395 and clopidogrel were dissolved in DMSO solution, which was used as the vehicle. To examine the role of RhoA/ROCK2 in CM, animals were injected i.p. with the ROCK2 inhibitor fasudil (30 mg/kg; Selleck, TX, USA), which was diluted with saline. Fasudil was administered in the same manner as MRS2395 and clopidogrel. The concentrations and drug delivery methods of MRS2395, clopidogrel, and fasudil were selected based on previous research [19, 29–32].

### BV-2 cell culture and treatment

BV-2 cells were obtained from the China Center for Type Culture Collection (Wuhan, China) and were cultured in Dulbecco’s modified Eagle’s medium supplemented with F12 (DMEM/F12) (Gibco, NY, USA) containing 10% fetal bovine serum (FBS) (Gibco, NY, USA) and 1% penicillin/streptomycin (PS) (HyClone, UT, USA), and were incubated at 37 °C under 5% CO_2_. Cells were seeded in 100 mm culture dishes (1 × 10^6^ cells/dish for Western blotting), 24-well-culture plates (3 × 10^4^ cells/well for immunofluorescence assays and 10^5^ cells/well for ELISA), and serum-starved for 24 h before each experiment. First, BV-2 cells were stimulated with LPS (1 μg/ml, Sigma–Aldrich, MO, USA) for 0, 2, 4, 8, 16, and 24 h to assess the most effective time point of this agent. Then, cells were preincubated with P2Y12R antagonists, MRS2395 (20 μM, Sigma–Aldrich, MO, USA) and clopidogrel (0.18 μM and 1.8 μM; Selleck, TX, USA) [33, 34], for 2 h, followed by LPS stimulation for 24 h.

### Sensory sensitivity testing

All behavioral experiments were conducted in low-light conditions, between 9:00 and 15:00. Animals were habituated to the testing chambers for 2 days prior to behavioral tests. The experimenter was blinded to the specific experimental groups of animals and the behavioral data analysis. Although both fasudil and NTG are vasodilators, no significant hypotensive signs were observed during the experiments.

Headache is a hallmark of migraine pain, and abnormal skin pain is a manifestation of hypersensitivity [35], thus, we examined both head-specific (periorbital) allodynia and hindpaw hyperalgesia to reflect the characteristics of the CM animal model. For periorbital mechanical sensitivity, the mice were placed in 4 oz. paper cups, to which they had been previously habituated, such that only the head poked out and prevented the mice from turning in the cups. Then, the periorbital region, including the caudal regions of eyes to approximately the midline, was stimulated with an electronic von Frey monofilament (ElectrovonFrey 2391, IITC Inc., Woodland Hills, CA, USA). The bending force of the von Frey apparatus was ranged from 0 to 800 g, and the sensitivity of this apparatus is 0.1 g. The stimuli were gradually increased to investigate the response thresholds. Positive responses were defined as quick retraction of the head upon stimulation or scratching of the face with the ipsilateral forepaw. The pressure thresholds were recorded automatically, and at each site, the test was repeated three times with an interval of at least 1 min. For hindpaw sensitivity, mice were placed in acrylic cages with wire grid floors in a quiet room 30 min before the start of testing, then the tip was applied perpendicularly to the central area of the plantar surface of the animal hindpaw with a gradual increase in pressure. Positive responses were defined as lifting or shaking of the paw upon stimulation [36]. After paw withdrawal, the pressure thresholds were automatically recorded, and the final values for the response was obtained by averaging three measurements.

To test thermal nociceptive responses, the paw withdrawal latencies in responding to noxious heat stimuli were measured using a plantar test apparatus (Techman PL-200, Chengdu, China), which focused radiant light on the plantar surface of the hindpaw. The withdrawal latencies were recorded automatically once the mice lifted their paws. Each animal was tested three times at intervals of at least 5 min. The heat intensity was calibrated to produce an average withdrawal latency of 8–10 s in normal animals. To prevent tissue injury, the maximum cut-off time was set at 20 s [37]. The schedule of the experimental timeline was as follows: animals were habituated to the test rack for 30 min on the testing day, and the basal mechanical and thermal allodynia were then measured. After behavioral tests, mice were treated with MRS2395, clopidogrel, fasudil, and vehicle, followed by NTG injection 30 min later, and the acute mechanical and thermal responses were assessed 2 h later. The process was repeated every other day for 9 days.

### Western blot analysis

For c-fos detection, TNC tissues were collected 2 h after NTG or vehicle administration, while for other targets, TNC tissues were collected 24 h after NTG administration. Mice were deeply anesthetized with 10% chloral hydrate, and the TNC was removed immediately, rinsed in cold phosphate-buffered saline (PBS), and stored at − 80 °C. Tissues were homogenized in cold RIPA lysis buffer (Beyotime, Shanghai, China) containing phenylmethylsulfonyl fluoride (PMSF, Beyotime, Shanghai, China) for 1 h at 4 °C and were then centrifuged at 12,000 g for 15 min at 4 °C. Protein content was determined using a BCA protein assay kit (Beyotime, Shanghai, China). Equal amounts of protein samples (50 μg) were separated by 10% SDS-polyacrylamide gel (Beyotime, Shanghai, China) electrophoresis, and transferred to PVDF membranes. Then, membranes were blocked in TBST containing 5% fat-free milk solution for 2 h at room temperature, followed by incubation with the following antibodies overnight at 4 °C: rabbit anti-P2Y12R (1:1000, Abcam, Cambridge, MA, USA), rabbit anti-CGRP (1:5000, Abcam, Cambridge, MA, USA), rabbit anti-c-fos (1:5000, Novus, CO, USA), mouse anti-iNOS (1:200, Santa Cruz, CA, USA), rabbit anti-RhoA (1:500, Proteintech, China), mouse anti-ROCK2 (1:200, Santa Cruz, CA, USA), and mouse anti-β-actin (1:2000, ZSGB-BIO, China). Membranes were then washed with TBST, and incubated with horseradish peroxidase-conjugated secondary antibodies (goat anti-rabbit, 1:9000; goat anti-mouse, 1:5000; ZSGB-BIO, China) for 1 h at room temperature. Subsequently, enhanced chemiluminescence reagent (Advansta Inc., USA) was used to detect signals, and the blots were visualized and analyzed using an image analysis system (Fusion, Germany). The band densities were normalized to those of the corresponding β-actin loading controls. Each Western blot analysis was repeated at least six times, and consistent results were obtained.

To determine active RhoA (membrane GTP-RhoA) expression, we separated the cytosolic and membrane fractions as described in a previous report [38]. After the TNC homogenate was centrifuged, the supernatant was ultracentrifuged at 100,000 g for 60 min at 4 °C. The obtained supernatant was considered the cytoplasmic fraction. The pellet was resuspended in ice-cold Tris buffer and ultracentrifuged again. The supernatant was removed, and then the pellet was resuspended in ice-cold Tris buffer, and stored as the plasma membrane fraction. Equal amounts of cytosolic and membrane proteins were analyzed using Western blotting as described above. The expression of GTP-RhoA was compared with that of total RhoA.

To investigate the expression of P2Y12R and inducible nitric oxide synthase (iNOS) in BV-2 cells, we collected cellular protein after LPS stimulation. Cells were washed with cold PBS three times and lysed in cold RIPA buffer containing PMSF for 30 min at 4 °C, and the protein concentration was then detected by a BCA protein assay kit. The following Western blot procedures were performed as described above. Each Western blot analysis was repeated in three independent experiments.

### Immunofluorescence staining

Animals were anesthetized and transcardially perfused with 30 ml of cold PBS (pH 7.4), followed by 50 ml of cold 4% paraformaldehyde (PFA) (dissolved in PBS solution). The whole brain and cervical spinal cord (C1-C2) were removed and postfixed in 4% PFA at 4 °C overnight. The medullary segment containing the TNC between + 1 and − 3 mm from the obex was removed and transferred sequentially to 20% and 30% sucrose/PBS solutions until it sank. Tissues were frozen and sectioned on a cryostat (Leica) at a thickness of 10 μm. Sections were blocked with 5% normal goat/donkey serum containing 0.3% Triton X-100 for 1 h at room temperature, and were then incubated with the following primary antibodies overnight at 4 °C: rabbit anti-P2Y12R (1:500, Anaspec Inc., MA, USA), rabbit anti-Iba1 (1:500, Wako Chemicals, Tokyo, Japan), goat polyclonal anti-Iba1 (1:100, Abcam, Cambridge, MA, USA), mouse anti-GFAP (1:500, Abcam, Cambridge, MA, USA), mouse anti-NeuN (1:500, Novus, CO, USA), mouse anti-CGRP (1:100, Santa Cruz, CA, USA), and rabbit anti-c-fos (1:5000, Novus, CO, USA). After washing in PBS, sections were incubated with fluorescence-conjugated secondary antibodies (Alexa Fluor 488 and Alexa Fluor 555, 1:500, Beyotime, China) for 2 h at room temperature. Nuclei were stained with 4′,6-diamidino-2-phenylindole (DAPI) (Beyotime, China) at room temperature for 10 min. Sections were mounted, and images were acquired with a confocal microscope (LSM800, Zeiss). The Fluorescence signal intensity was quantified using image analysis software (Image-Pro Plus 6.2, Media Cybernetics). To quantify P2Y12R-, c-fos-, and Iba-1-positive cell profiles in the TNC, six sections per mouse from four mice were randomly selected from each group. An image in a square (320 × 320 μm^2^) centered on the superficial layer of the TNC was acquired with a × 200 objective, and all immunoreactive cells in the area were counted with image analysis software (Image-Pro Plus 6.2, Media Cybernetics). To analyze microglial morphological features, 8–10 visual fields per mouse were investigated. The total and mean length of microglial processes emanating directly from the cell body were determined by Neuron J (an ImageJ plug-in) to facilitate the tracing and quantification of elongated structures in the 2D images [18]. Data are presented as mean ± SEM. Data were analyzed by one-way analysis of variance (ANOVA) with Tukey’s test as post hoc comparisons. A difference was considered significant if *p* < 0.05.

### Immunocytochemistry

We also examined the level of P2Y12R in BV-2 cells and its effects on BV-2 microglial morphological changes and iNOS production using immunocytochemistry. BV-2 cells (3 × 10^4^ cells/well in 24-well plates) were seeded on glass coverslips, preincubated with MRS2395 (20 μM) and clopidgrel (1.8 μM) for 2 h followed by LPS for 24 h, cover slips were then washed in PBS and fixed in 4% PFA for 30 min at room temperature. Coverslips were blocked with 5% goat/donkey serum containing 0.3% Triton X-100 for 30 min at room temperature, and were then incubated with following primary antibodies overnight at 4 °C: rabbit anti-P2Y12 (1:500, Anaspec Inc), goat anti-Iba1 (1:200, Abcam), and mouse anti-iNOS (1:50, Santa Cruz). The next day, coverslips were washed and incubated with fluorescence-conjugated secondary antibodies (Alexa Fluor 488 and Alexa Fluor 555, 1:500, Beyotime, China) for 2 h at room temperature, and were then stained with DAPI for 10 min. After washing, coverslips were mounted and observed with a confocal microscope (LSM800, Zeiss).

### Enzyme-linked immunosorbent assay

Culture medium from BV-2 cells, incubated with vehicle, LPS, or MRS2395 and clopidogrel, was collected and centrifuged at 1000 rpm for 5 min at 4 °C, and the supernatant was stored at − 80 °C until assay. Interleukin-1β (IL-1β) and tumor necrosis factor-α (TNF-α) released into the culture supernatants were measured using commercial mouse IL-1β and TNF-α enzyme-linked immunosorbent assay (ELISA) kits (Boster Biological Technology, Wuhan, China), according to the manufacturer’s instructions. Briefly, samples and protein standards were added to 96-well ELISA plates, and were then interacted with biotinylated anti-IL-1β or anti-TNF-α antibody. After washing, the prepared solution containing avidin, horseradish peroxidase-conjugated complex was added to the plates, and substrate solution (TMB) was then added. Finally, we added stop solution to terminate the reaction. The optical density was measured by a microplate reader at 450 nm, and the concentrations of IL-1β and TNF-α were calculated from the standard curve.

### Statistical analysis

SPSS 22.0 software (IBM Corp, Armonk, NY, USA) was used to conduct statistical analyses. All data are presented as mean ± SEM. Differences between two groups were analyzed by Student’s *t* test. Changes in the measured protein expression, calcitonin gene-related peptide (CGRP) immunoreactivity, and numbers of c-fos and Iba-1-positive cells were determined by one-way ANOVA followed by individual post hoc comparisons (Tukey’s test). Behavioral data were analyzed using two-way ANOVA with a Bonferroni post hoc test. A difference was considered statistically significant if *p* < 0.05.

## Results

### Upregulation of microglial P2Y12R in the TNC after chronic NTG administration

**Fig. 1:**
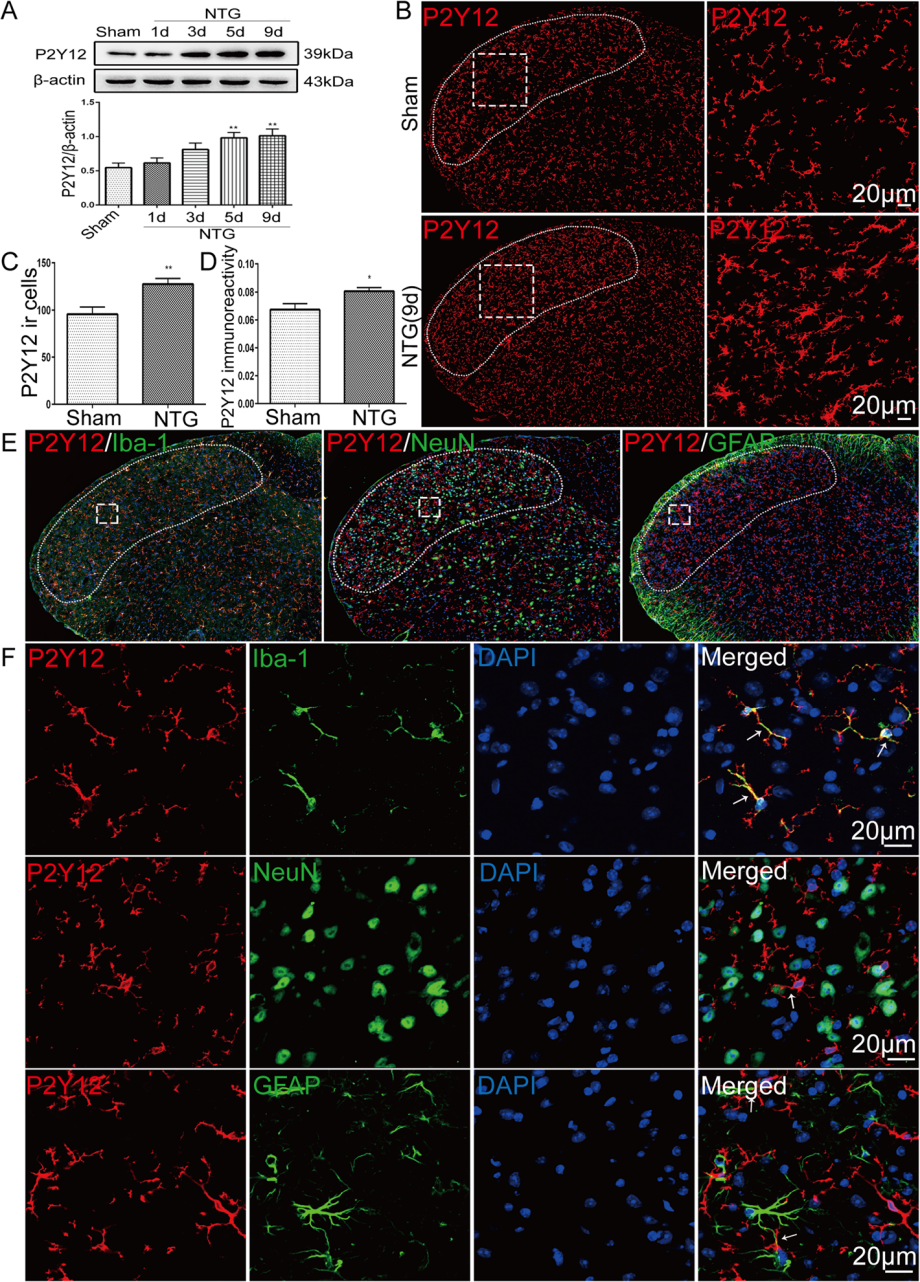
Chronic NTG administration increased microglial P2Y12R expression in the TNC. **a** Representative Western blot showing P2Y12R protein levels in the TNC following NTG injection. The band intensities are presented relative to those of β-actin. Data are presented as mean ± SEM; *n* = 6 per group; **p < 0.01 compared with the sham group. **b** Representative immunofluorescence images of P2Y12R in the TNC after NTG administration. The left graphs show the TNC regions, which marked by the white dotted line frames. The right graphs show higher magnification images of P2Y12R in the TNC. Scale bar: 20 μm. **c**, **d** Quantitative analysis of the number of P2Y12R-immunoreactive (ir) cells (**c**) and the P2Y12R immunoreactive area in the TNC (**d**) in a 320 × 320 μm^2^ visual field of each section per mouse after NTG injection. Data are presented as mean ± SEM; *n* = 4 per group; six sections from each mouse; *p < 0.05 and **p < 0.01 compared with the sham group. **e**, **f** Double-immunofluorescence labeling of P2Y12R with iba-1, NeuN, and GFAP in the TNC after NTG administration. The graphs in **e** show the TNC location which is marked by the white dotted line frame. The graphs in **f** show higher magnification images. Scale bar: 20 μm

We used Western blotting to detect the effect of chronic NTG administration on the expression of P2Y12R in the TNC over time (*n* = 6 per group). We found that recurrent NTG injection induced a time-dependent increase in P2Y12R protein expression in the immunoblot analyses (Fig. 1a). The P2Y12R protein levels were significantly increased on day 5 (*p* < 0.01), and peaked on day 9 (*p* < 0.01) after NTG injection. We also performed immunofluorescence for P2Y12R using a specific antibody (*n* = 4 per group) (Fig. 1b). Consistent with the Western blot analysis results, both the staining intensity of P2Y12R (*p* < 0.05) and the number of P2Y12R-positive cells (*p* < 0.01; NTG 9 d, 127.75 ± 5.97/section; Sham, 95.7 ± 7.68/section) in the TNC were markedly increased following chronic NTG administration compared with the values in the control group (Fig. 1c, d). To localize P2Y12R to specific cell types in the TNC, we used double-labeling studies with P2Y12 protein and cell type-specific markers (Ibal-1 to label microglia, NeuN to label neurons, and GFAP to label astrocytes). We observed that P2Y12R-positive cells were colocalized with Iba-1 (Fig. 1e, f). From these results, we concluded that P2Y12R is upregulated in TNC microglia in the NTG-induced CM model.

### Blockage of P2Y12R activity attenuated CM-associated mechanical allodynia and thermal hyperalgesia

**Fig. 2:**
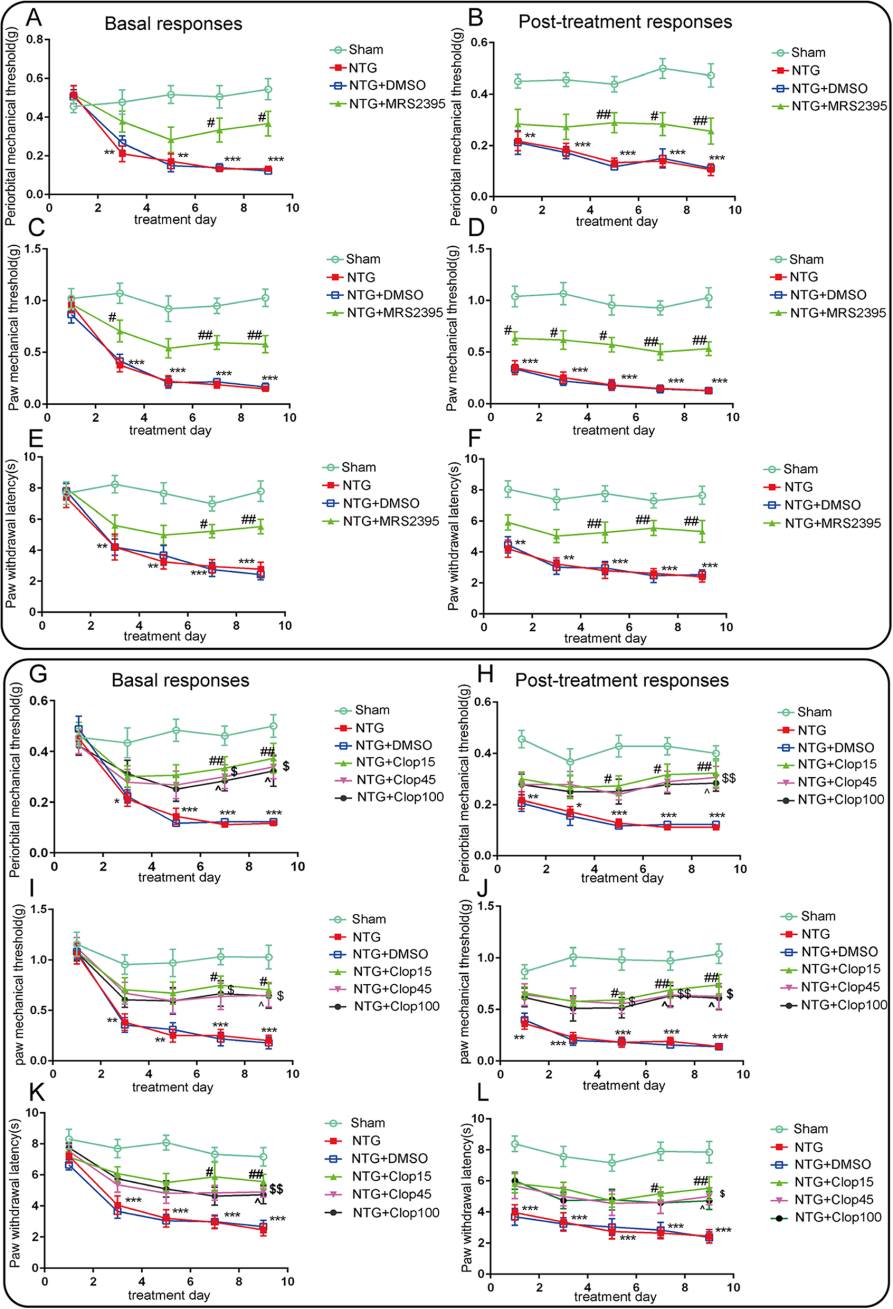
The P2Y12R antagonists MRS2395 and clopidogrel prevented NTG-induced mechanical allodynia and thermal hyperalgesia. Repeated treatment with MRS2395 blocked basal periorbital mechanical allodynia (**a**) and hindpaw mechanical (**c**) and thermal hyperalgesia (**e**). Post-treatment responses including periorbital mechanical allodynia (**b**) and hindpaw mechanical (**d**) and thermal hyperalgesia (**f**) were assessed 2 h after NTG administration. Data are presented as mean ± SEM; *n* = 10 per group; two-way ANOVA and Bonferroni post hoc analysis; **p < 0.01 and ***p < 0.001 compared with the sham group; ^#^*p* < 0.05 and ^##^*p* < 0.01 compared with the NTG group. Similarly, i.p. treatment with clopidogrel (15, 45, and 100 mg/kg; *n* = 10 per group) also showed an analgesic effect in mice with basal and post-treatment mechanical allodynia (**g**–**j**) and thermal hyperalgesia (**k**, **l**) after NTG injection. Values are presented as mean ± SEM; two-way ANOVA and Bonferroni post hoc analysis; *p < 0.05, **p < 0.01, and ***p < 0.001 compared with the sham group; ^#,$,^^*p* < 0.05 and ^##,$$,^^^*p* < 0.01; 15, 45 and 100 mg/kg-treated groups, respectively, compared with the NTG group

Consistent with previous studies, we observed that in mice treated with NTG or NTG with DMSO, the thresholds of paw and periorbital withdrawal from a mechanical stimulus and the paw withdrawal latencies to noxious heat were gradually decreased in a time-dependent manner both before and 2 h after NTG injection (Fig. 2a–l), specifically, chronic NTG administration produced progressive and sustained basal hypersensitivity as well as acute allodynia. To evaluate whether P2Y12R in TNC microglia plays a critical role in NTG-induced pain behavior, we i.p. injected the P2Y12R selective antagonist MRS2395 (1.5 mg/kg) prior to NTG administration every other day for 5 days (*n* = 10 per group). We found that chronic treatment with MRS2395 completely attenuated the basal hypersensitivity (Fig. 2a, c, e) and acute hyperalgesia (Fig. 2b, d, f). To confirm whether the analgesic effect resulting from P2Y12R inhibition is relevant to clinical practice, we treated mice i.p. with the peripherally active P2Y12R inhibitor clopidogrel (15, 45, and 100 mg/kg) prior to NTG injection five times (*n* = 10 per group). The basal and post-treatment mechanical and thermal thresholds were markedly increased compared with those in the NTG group (Fig. 2g–l), and significant changes were observed on day 7 (15 mg/kg), and on day 9 (15, 45, and 100 mg/kg). Thus, P2Y12R function contributes to the development of CM-like pain hypersensitivity.

### Inhibition of P2Y12R reduced CGRP and c-fos expression in the TNC in recurrent NTG-treated mice

**Fig. 3:**
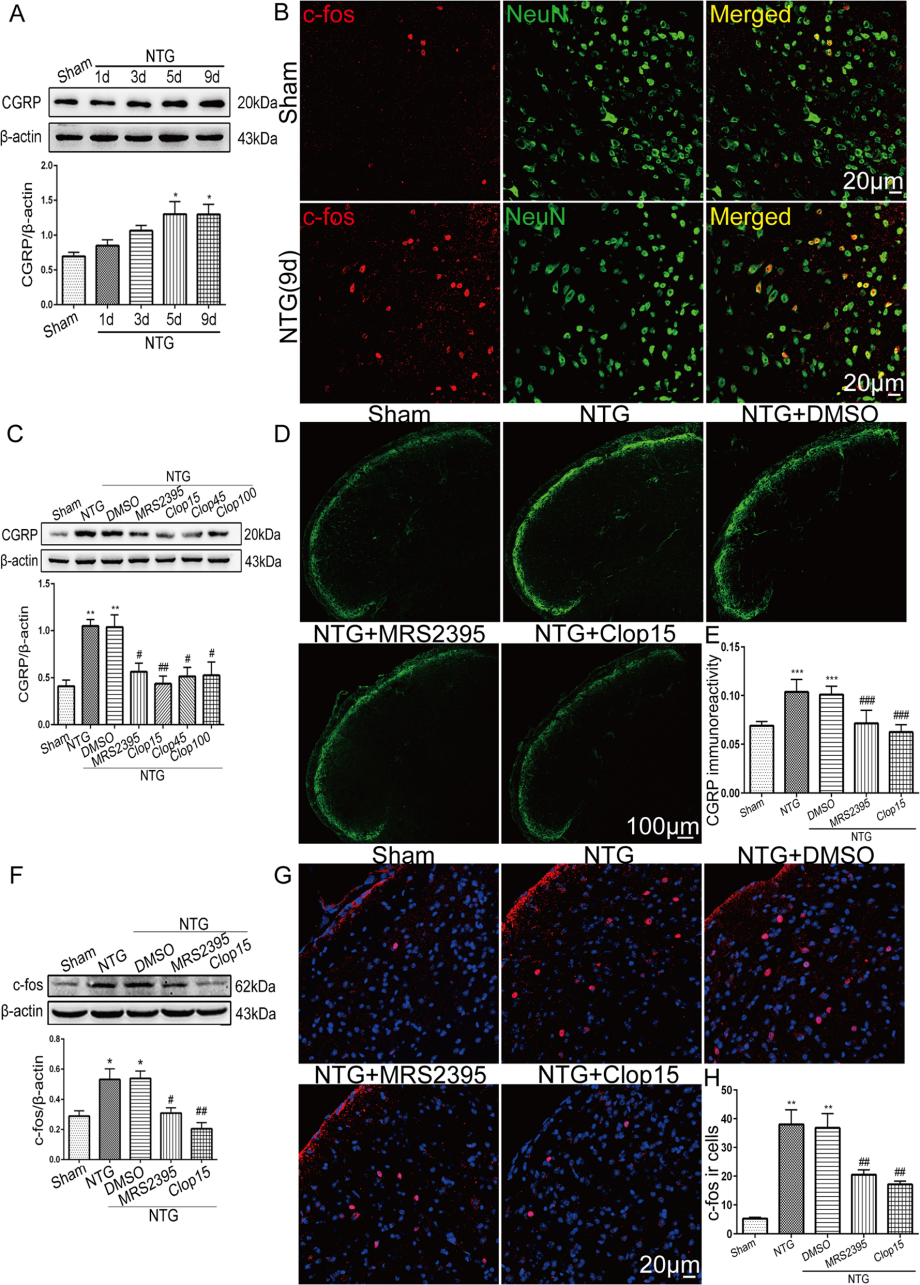
Inhibition of P2Y12R function reduced NTG-induced CGRP and c-fos expression in the TNC. **a** Representative western blots showing the changes in CGRP expression over time in the TNC after NTG administration. The band intensities are presented relative to those of β-actin. Quantification of CGRP protein levels showed a significant increase on days 5 and 9 after NTG injection. Data are presented as mean ± SEM; *n* = 6 per group; *p < 0.05 compared with the sham group. **b** Double immunofluorescence staining images of c-fos with NeuN in sham and NTG injected mice. Scale bar: 20 μm. **c**, **f** Western blots showing that the P2Y12R inhibitors MRS2395 and clopidogrel partially suppressed the increase in CGRP (**c**) and c-fos (**f**) protein levels in the TNC; clopidogrel (15 mg/kg) showed the most significant inhibitory effect on CGRP (**c**). The band intensities are presented relative to those of β-actin. Values are presented as mean ± SEM; *n* = 6 per group; *p < 0.05 and **p < 0.01 compared with the sham group; ^#^*p* < 0.05 and ^##^*p* < 0.01 compared with the NTG group. **d**, **g** Immunofluorescence staining images of CGRP (**d**) and c-fos (**g**) in the TNC. Scale bars: 100 μm for CGRP, 20 μm for c-fos. **e**, **h** Quantitative analysis of CGRP immunoreactivity (**e**) and c-fos-ir cells (**h**) in the TNC after NTG injection. The results show changes consistent with the Western blot analysis results. Data are presented as mean ± SEM; *n* = 4 per group; six sections from each mouse; **p < 0.01 and ***p < 0.001 compared with the sham group; ^##^*p* < 0.01 and ^###^*p* < 0.001 compared with the NTG group

CGRP, a critical endogenous mediator of migraine, is involved in the occurrence and maintenance of migraine [39]. To determine whether the NTG induced CM-like hypersensitivities correspond to the release of CGRP, we examined the CGRP protein level at different time points using Western blotting (*n* = 6 per group) (Fig. 3a). Statistically significant upregulation of CGRP was observed on day 5 (*p* < 0.05) and on day 9 (p < 0.05) after NTG injection. We also measured the c-fos level in the TNC, which is a reliable marker of neuronal activation. Using immunofluorescence, we found that the number of c-fos immunoreactive cells in the TNC in the NTG administration group was increased compared with that in the sham group (Fig. 3b). These findings indicate that CGRP and c-fos play a critical role in NTG-induced CM associated hyperalgesia.

To determine whether P2Y12R activity affects CGRP and c-fos production, we inhibited P2Y12R by systemic administration of its antagonists MRS2395 (1.5 mg/kg) and clopidogrel (15, 45, 100 mg/kg) (*n* = 6 per group). The results showed that the protein level of CGRP in the TNC was significantly decreased by MRS2395 (*p* < 0.05) and clopidogrel (*p* < 0.01 for 15 mg/kg; *p* < 0.05 for 45 mg/kg; *p* < 0.05 for 100 mg/kg) compared with the corresponding level in the NTG treated group (Fig. 3c). The density of CGRP-immunoreactive fibers in the TNC during NTG administration was also reduced by MRS2395 (*p* < 0.001) and clopidogrel (15 mg/kg) (*p* < 0.001) (Fig. 3d, e). Consistent with the alterations in CGRP, the protein level of c-fos in the TNC was significantly decreased by MRS2395 (*p* < 0.05) and clopidogrel (15 mg/kg) (*p* < 0.01) (Fig. 3f). The numbers of c-fos immunoreactive cells in the superficial layer of the TNC were also decreased in the groups treated with MRS2395 (*p* < 0.01) and clopidogrel (*p* < 0.01) (Fig. 3g, h).

### P2Y12R affected NTG-induced microglial morphological alterations and iNOS production in the TNC

**Fig. 4:**
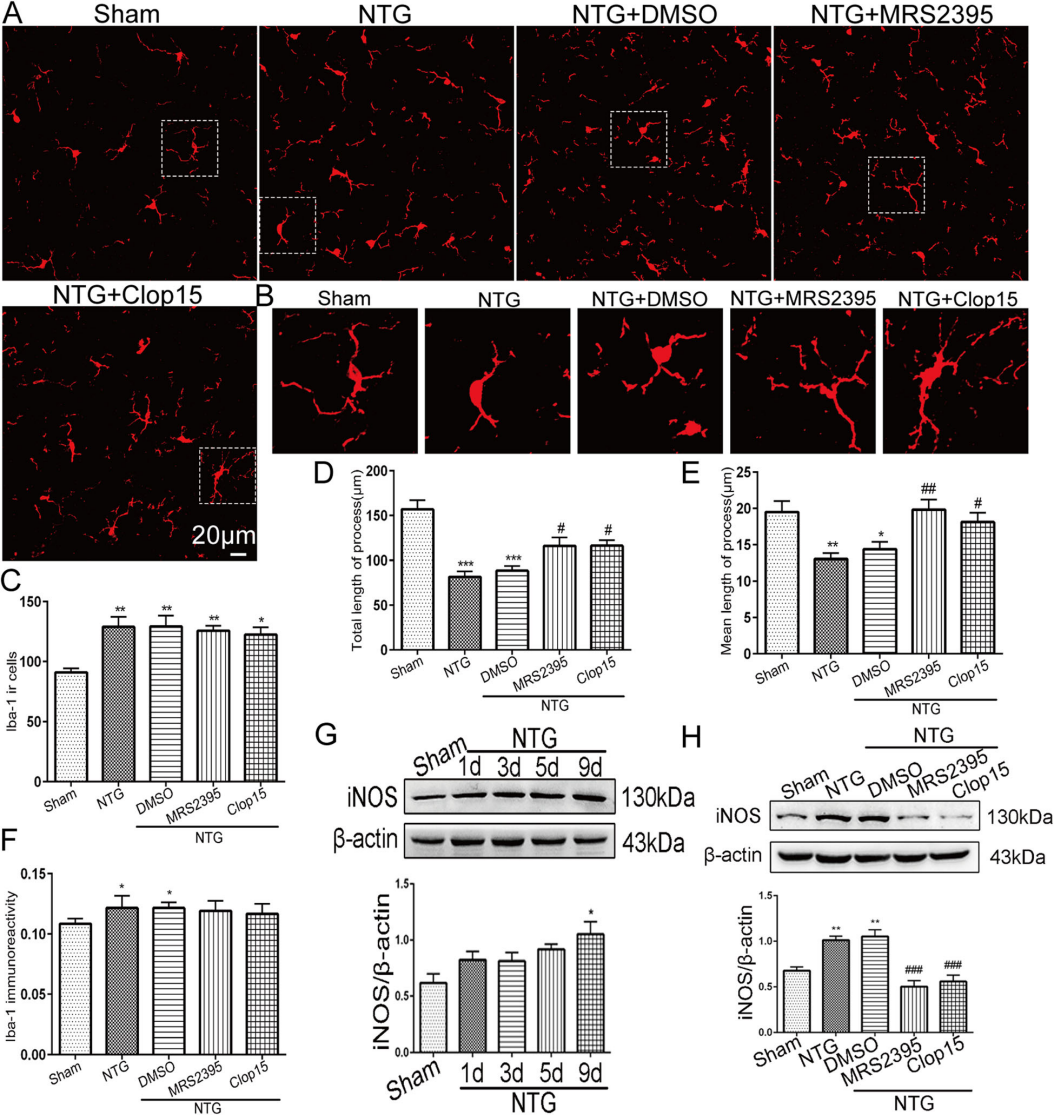
P2Y12R affected NTG-induced microglial morphological alterations and iNOS production in the TNC. **a** Iba-1 immunostaining in the TNC following NTG, MRS2395, or clopidogrel administration. Scale bar: 20 μm. **b** Magnified images of Iba-1 in the area enclosed in the white dotted line frame in a. **c**, **f** Quantification of Iba-1-positive cells (**c**) and Iba-1 immunoreactivity (**f**) in the TNC. Data are presented as mean ± SEM; *n* = 4 per group; six sections from each mouse; *p < 0.05, **p < 0.01 compared with the sham group. **d**, **e** Quantitative analysis of the total length of initial processes (**d**) and the mean length of initial processes per microglia (**e**). Data are presented as mean ± SEM. A total of 340 cells (71 cells in the sham group, 68 cells in the NTG group, 62 cells in the NTG + DMSO group, 67 cells in the NTG + MRS2395 group, 72 cells in the NTG + Clop15 (15 mg/kg) group) were analyzed. *p < 0.05, **p < 0.01, and ***p < 0.001 compared with the sham group; ^#^*p* < 0.05 and ^##^*p* < 0.01 compared with the NTG group. **g** Representative immunoblots of iNOS expression in the TNC at various time points after NTG administration. The band intensities are presented relative to those of β-actin. Quantification of iNOS expression indicated a significant increase on day 9 after NTG injection. Data are presented as mean ± SEM; *n* = 6 per group; *p < 0.05 compared with the sham group. **h** Western blot showing that the P2Y12R inhibitors MRS2395 and clopidogrel (15 mg/kg) reduced the production of iNOS induced by NTG in the TNC. Values are presented as mean ± SEM; *n* = 6 per group; **p < 0.01 compared with the sham group; ^###^*p* < 0.001 compared with the NTG group

We used immunofluorescence to detect alterations in microglia in the TNC in the NTG-induced CM model. After recurrent NTG administration, the staining intensity of Iba-1 (*p* < 0.05) and the number of Iba-1 immunoreactive cells (*p* < 0.01) in the TNC were markedly increased (Fig. 4a, c, f), and features of activated microglia, including hypertrophic somata and shortened processes, were observed (Fig. 4b, d, e). Next, the results of systemic treatment with MRS2395 and clopidogrel (*n* = 4 per group) prior to NTG revealed that neither antagonist reduced the Iba-1 staining intensity or number of positive cells compared with the corresponding values in the NTG group (Fig. 4a, c, f). However, the total length (*p* < 0.05, NTG + MRS2395, 116.11 ± 9.387 μm; *p* < 0.05, NTG + Clop15, 116.31 ± 6.027 μm vs. NTG, 81.70 ± 6.162 μm) and mean length (*p* < 0.01, NTG + MRS2395, 19.85 ± 1.397 μm; *p* < 0.05, NTG + Clop15, 18.11 ± 1.285 μm; vs. NTG, 13.05 ± 0.794 μm) of the microglial processes were significantly increased compared with those in the NTG-treated group (Fig. 4b, d, e). We also measured the expression of the microglial inflammatory factor, iNOS in the TNC during recurrent NTG injection (*n* = 6 per group), and found that the level of iNOS increased over time and peaked on day 9 (*p* < 0.01) (Fig. 4g). However, with the inhibition of P2Y12R activation via recurrent administration of MRS2395 and clopidogrel, iNOS expression in the TNC was significantly reduced in both groups (*p* < 0.001) (Fig. 4h). Thus, we concluded that P2Y12R plays a critical role in microglial activation in the TNC in the NTG-induced CM model.

### P2Y12R antagonists inhibited the activation of RhoA/ROCK2 in the TNC during NTG-induced CM mice

**Fig. 5:**
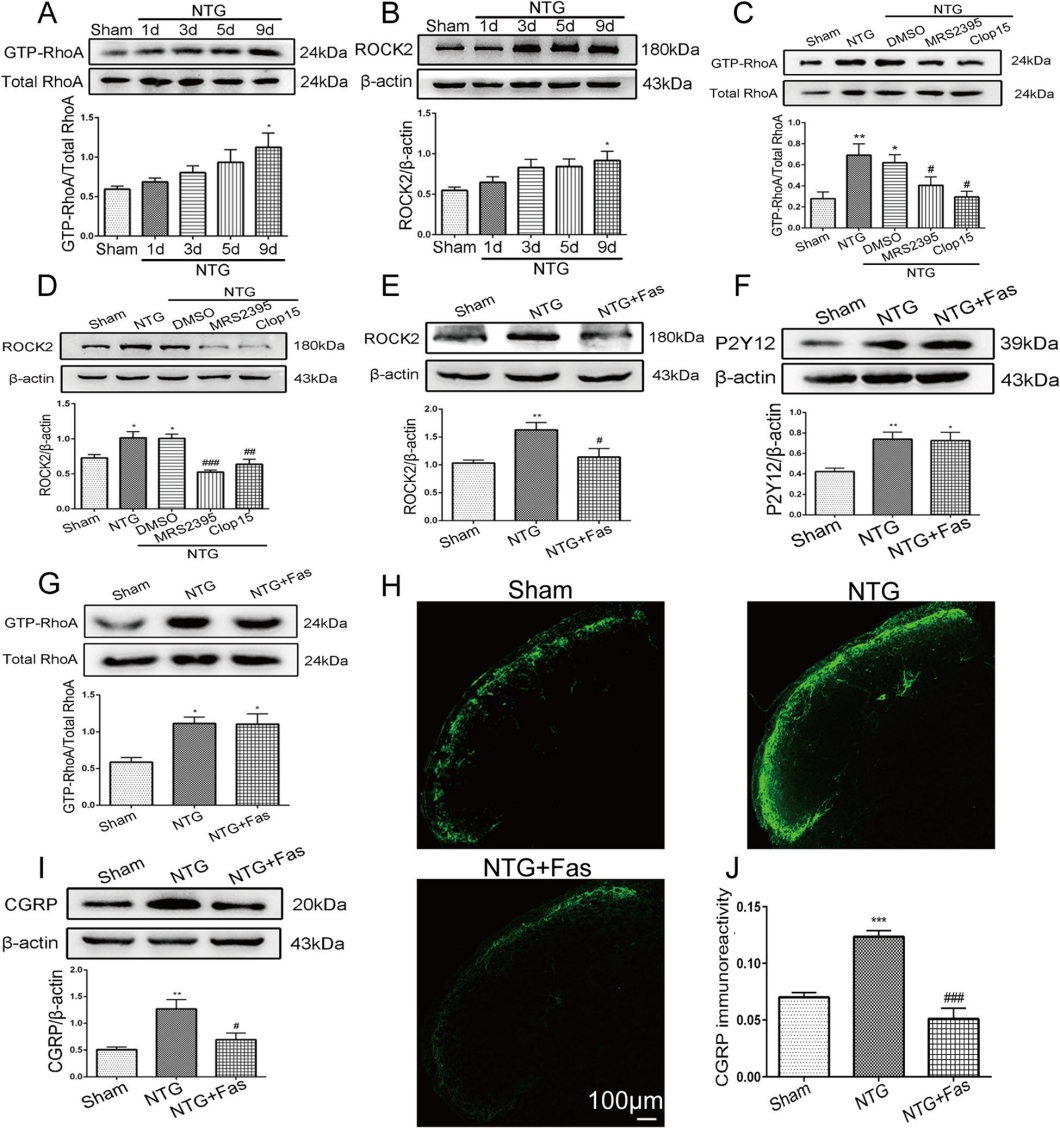
RhoA/ROCK2 signaling, which acts downstream of P2Y12R in the TNC, was increased following NTG administration and contributed to NTG-induced CGRP expression. **a**, **b** Representative immunoblots of GTP-RhoA and ROCK2 in the TNC at various time points after NTG administration. The band intensities of GTP-RhoA and ROCK2 are presented relative to those of total RhoA and β-actin, respectively. Quantitative analysis indicated a significant increase on day 9 after NTG injection. Data are presented as mean ± SEM; *n* = 6 per group; *p < 0.05 compared with the sham group. **c**, **d** Western blot showing that the P2Y12R inhibitors MRS2395 and clopidogrel attenuated the upregulation of GTP-RhoA and ROCK2 expression. Values are presented as mean ± SEM; *n* = 6 per group; *p < 0.05 and **p < 0.01 compared with the sham group, ^#^*p* < 0.05, ^##^*p* < 0.01, and ^###^*p* < 0.001 compared with the NTG group. The ROCK inhibitor fasudil reversed the upregulation of ROCK2 (**e**) in the TNC induced by NTG administration but had no effect on P2Y12R (**f**) and GTP-RhoA (**g**) expression in the TNC. Data are presented as mean ± SEM; *n* = 6 per group; *p < 0.05 and **p < 0.01 compared with the sham group, ^#^*p* < 0.05 compared with the NTG group. **h** Immunofluorescence staining images of CGRP in the TNC. Scale bar: 100 μm. **i** Representative immunoblots of CGRP in the TNC after NTG and fasudil administration. The band intensity is presented relative to that of β-actin. Data are presented as mean ± SEM; *n* = 6 per group; **p < 0.01 compared with the sham group; ^#^*p* < 0.05 compared with the NTG group. **j** Quantitative analysis of CGRP immunoreactivity in the TNC. Data are presented as mean ± SEM; *n* = 4 per group; six sections per mouse; ***p < 0.001 compared with the sham group; ^###^*p* < 0.001 compared with the NTG group

We measured the protein expression levels of P2Y12R downstream effectors, active RhoA (GTP-RhoA) and ROCK2, in the TNC after repeated NTG administration (1, 3, 5, and 9 days post injection) and observed that these protein levels gradually increased in a time-dependent manner and peaked on day 9 (*p* < 0.05, GTP-RhoA in NTG 9d, 1.13 ± 0.180 vs. Sham 0.59 ± 0.0391; *p* < 0.05, ROCK2 in NTG 9d 0.92 ± 0.113 vs. Sham 0.55 ± 0.412) (Fig. 5a, b). After repeated treatment with the P2Y12R antagonists MRS2395 and clopidogrel, we found a significant decrease in the GTP-RhoA (*p* < 0.05, NTG + MRS2395, 0.37 ± 0.623; *p* < 0.001, NTG + Clop15, 0.29 ± 0.054 vs. NTG, 0.69 ± 0.108) and ROCK2 (*p* < 0.05, NTG + MRS2395, 0.53 ± 0.029; *p* < 0.01, NTG + Clop15, 0.64 ± 0.069 vs. NTG, 1.02 ± 0.089) levels in the TNC (Fig. 5c, d). To further confirm the inhibitory effect of P2Y12R on RhoA/ROCK2, we administered the ROCK2 inhibitor fasudil (30 mg/kg) i.p. every other day five times, and observed that the expression of only the ROCK2 protein (*p* < 0.05) was inhibited (Fig. 5e); however, the levels of P2Y12R and GTP-RhoA in the TNC did not decrease compared with those in the NTG group (Fig. 5f, g). These results reveal that P2Y12R regulates RhoA/ROCK activity in the TNC.

### Inhibition of ROCK2 activity suppressed NTG-induced CGRP level in the TNC and reduced CM-associated pain behavior

**Fig. 6:**
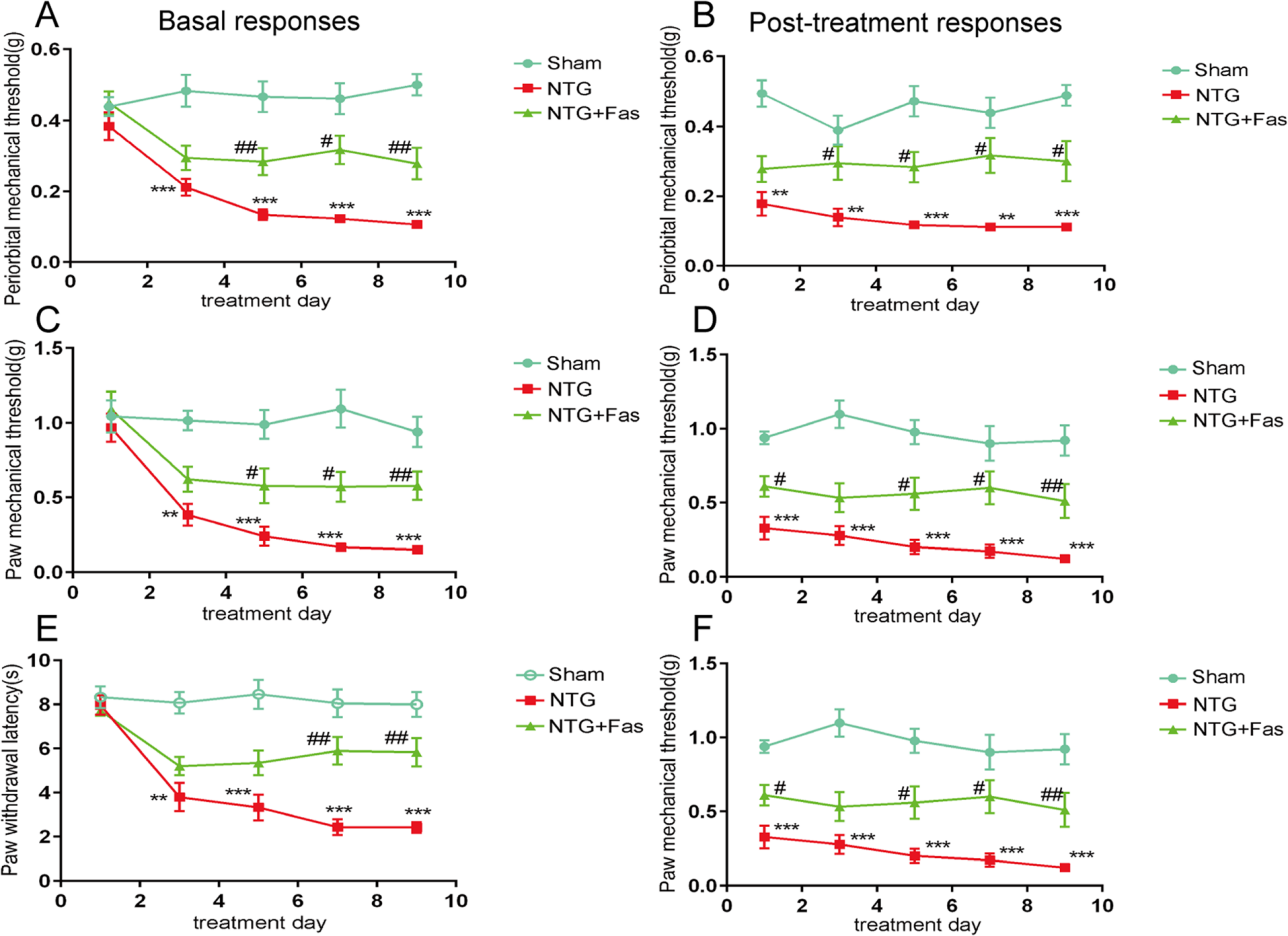
Inhibition of ROCK2 activity attenuated NTG induced CM-like behaviors. Repeated treatment with the ROCK2 antagonist fasudil significantly blocked NTG-induced basal periorbital mechanical allodynia (**a**) and hindpaw mechanical (**c**) and thermal hyperalgesia (**e**). Post-treatment responses, including periorbital mechanical allodynia (**b**) and hindpaw mechanical (**d**) and thermal hyperalgesia (**f**), were assessed 2 h after NTG administration. Data are presented as mean ± SEM; *n* = 10 per group; two-way ANOVA and Bonferroni post hoc analysis; **p < 0.01 and ***p < 0.001 compared with the sham group; ^#^*p* < 0.05 and ^##^*p* < 0.01 compared with the NTG group

To confirm whether the upregulation of RhoA/ROCK2 in the TNC in CM mice contributes to the mechanism of CM, we administered fasudil to assess its effect on CM-like pain behaviors and CGRP expression. We first performed Western blot and immunofluorescence to detect the inhibitory effect of fasudil on the expression of CGRP in the TNC during recurrent NTG administration. We found that the density of immunoreactive fibers for CGRP (*p* < 0.001) and the protein level of CGRP (*p* < 0.05) were significantly reduced by the ROCK2 inhibitor (Fig. 5h–j). The behavioral results showed that acute allodynia was completely abrogated (Fig. 6b, d, f), and that basal hyperalgesia was also markedly attenuated on days 5, 7, and 9 after fasudil treatment (Fig. 6a, c, e). These data indicate that RhoA/ROCK2, downstream of P2Y12R, is involved in the development of CM.

### ROCK2 activity affected morphological changes of microglia and iNOS expression in the TNC following NTG injection

**Fig. 7:**
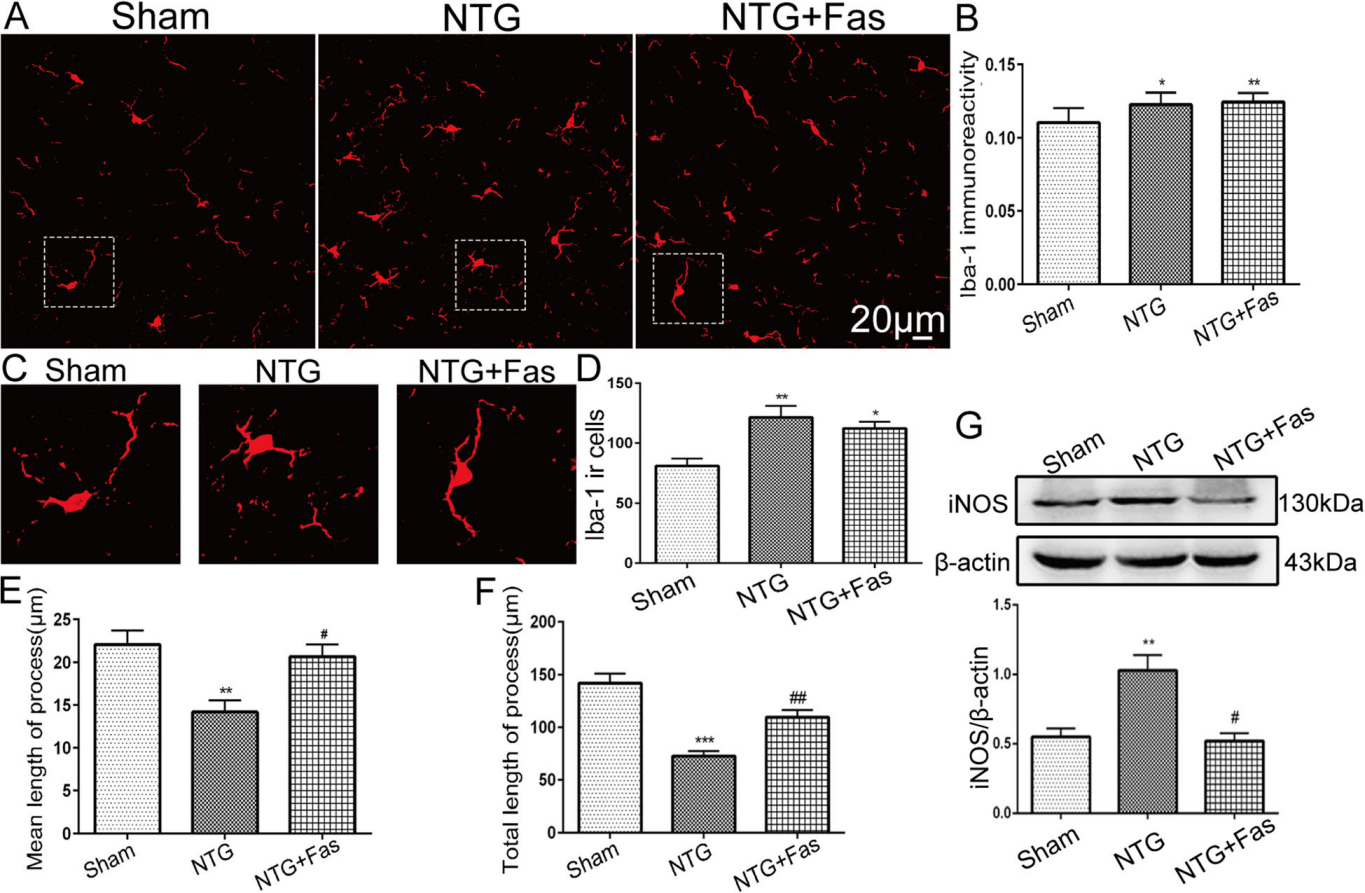
ROCK2 activity affected microglial morphological changes and iNOS expression in the TNC following NTG injection. **a** Iba-1 immunostaining in the TNC following NTG and fasudil administration. Scale bar: 20 μm. **b**, **d** Quantification of Iba-1 immunoreactivity (**b**) and Iba-1-positive cells (**d**) in the TNC. Data are presented as mean ± SEM; *n* = 4 per group; six sections from each mouse; **p < 0.01 compared with the sham group. **c** Magnified images of Iba-1 in the area enclosed in the white dotted line frame in **a**. **e**, **f** Quantitative analysis of the mean length of initial processes (**e**) and the total length of initial processes (**f**) per microglia. Data are presented as mean ± SEM, and a total of 224 cells (75 cells in the sham group, 77 cells in the NTG group, 72 cells in the NTG + Fas group) were analyzed. **p < 0.01 and ***p < 0.001 compared with the sham group; ^#^*p* < 0.05 and ^##^*p* < 0.01 compared with the NTG group. **g** Representative immunoblots of iNOS showing that the ROCK2 inhibitor fasudil reduced the production of iNOS induced by NTG in the TNC. Values are presented as mean ± SEM; *n* = 6 per group; **p < 0.01 compared with the sham group; ^#^*p* < 0.05 compared with the NTG group

We confirmed that chronic NTG administration activated microglia, which displayed hypertrophic somata and shortened processes, and increased the number and staining intensity of microglia (Fig. 7a–d). After repeated fasudil administration, although the increase in the microglial cell number (112.13 ± 5.563 cells in the NTG + Fas group, 121.25 ± 9.691 cells in the NTG group, *p* = 0.66) and staining intensity (0.12 ± 0.002 in the NTG + Fas group, 0.12 ± 0.003 in the NTG group, *p* = 0.89) in the TNC following recurrent NTG administration was not suppressed (Fig. 7b, d), the total length (*p* < 0.01; NTG + Fas, 109.41 ± 6.995 μm vs. NTG, 72.45 ± 4.784 μm) and mean length (*p* < 0.01; NTG + Fas, 20.67 ± 1.41 μm vs. NTG, 14.20 ± 1.353 μm) of the microglial processes per microglia were increased compared with those in the NTG group (Fig. 7e, f). In addition, using Western blotting, we found that the NTG-induced iNOS upregulation in the TNC was significantly suppressed by recurrent fasudil treatment (*p* < 0.01) (Fig. 7g).

### Upregulation of P2Y12R and inflammatory cytokines in LPS-stimulated BV-2 cells

**Fig. 8:**
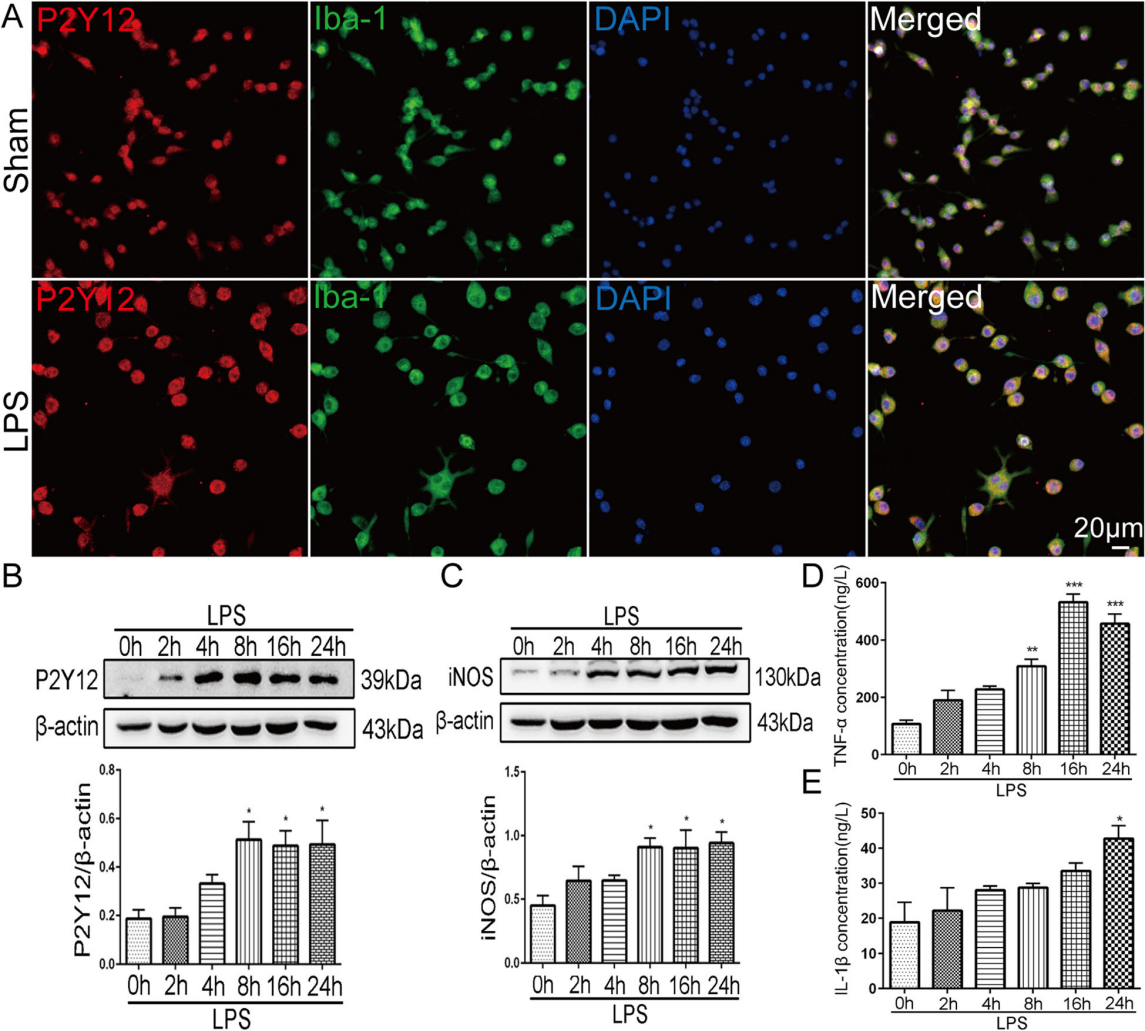
Upregulation of P2Y12R expression and inflammatory cytokine production in LPS-stimulated BV-2 cells. **a** Double immunofluorescence staining images of P2Y12R with Iba-1 in LPS-stimulated BV-2 cells. Scale bar: 20 μm. **b**, **c** Western blots showing the expression of P2Y12R and iNOS in BV-2 cells after LPS stimulation. The band intensity is presented relative to that of β-actin. Quantification of P2Y12R and iNOS protein levels indicated a significant increase at 8, 16, and 24 h after LPS stimulation. Data are presented as mean ± SEM of three independent experiments; *p < 0.05 compared with LPS 0 h. **d**, **e** ELISA of IL-1β and TNF-α release from LPS-stimulated BV-2 cells. Data are presented as mean ± SEM of three independent experiments; *p < 0.05, **p < 0.01, and ***p < 0.001 compared with LPS 0 h

To further confirm the effect of P2Y12R on microglial activation, we conducted experiments on BV-2 microglial cells. First, we performed double-labeling studies with P2Y12R and Iba-1, and observed that the P2Y12R-immunoreactive cells were colocalized with Iba1 (Fig. 8a). After LPS stimulation, the somata of BV-2 cells were hypertrophic (Fig. 8a), and the expression of P2Y12R increased over time. A statistically significant increase was observed at 8, 16, and 24 h (*p* < 0.05) (Fig. 8b). Then, we found that the trends in iNOS (*p* < 0.05 for 8 h, 16 h, and 24 h vs. 0 h) and TNF-α (*p* < 0.01 for 8 h; *p* < 0.001 for 16 h and 24 h vs. 0 h) upregulation were consistent with the P2Y12R level in LPS-stimulated BV-2 cells (Fig. 8c, d). In addition, the IL-1β level was markedly increased in BV-2 cells at 24 h (*p* < 0.05) after LPS incubation (Fig. 8e).

### Effect of P2Y12R on LPS-stimulated BV-2 microglial morphological changes and inflammatory cytokines production

**Fig. 9:**
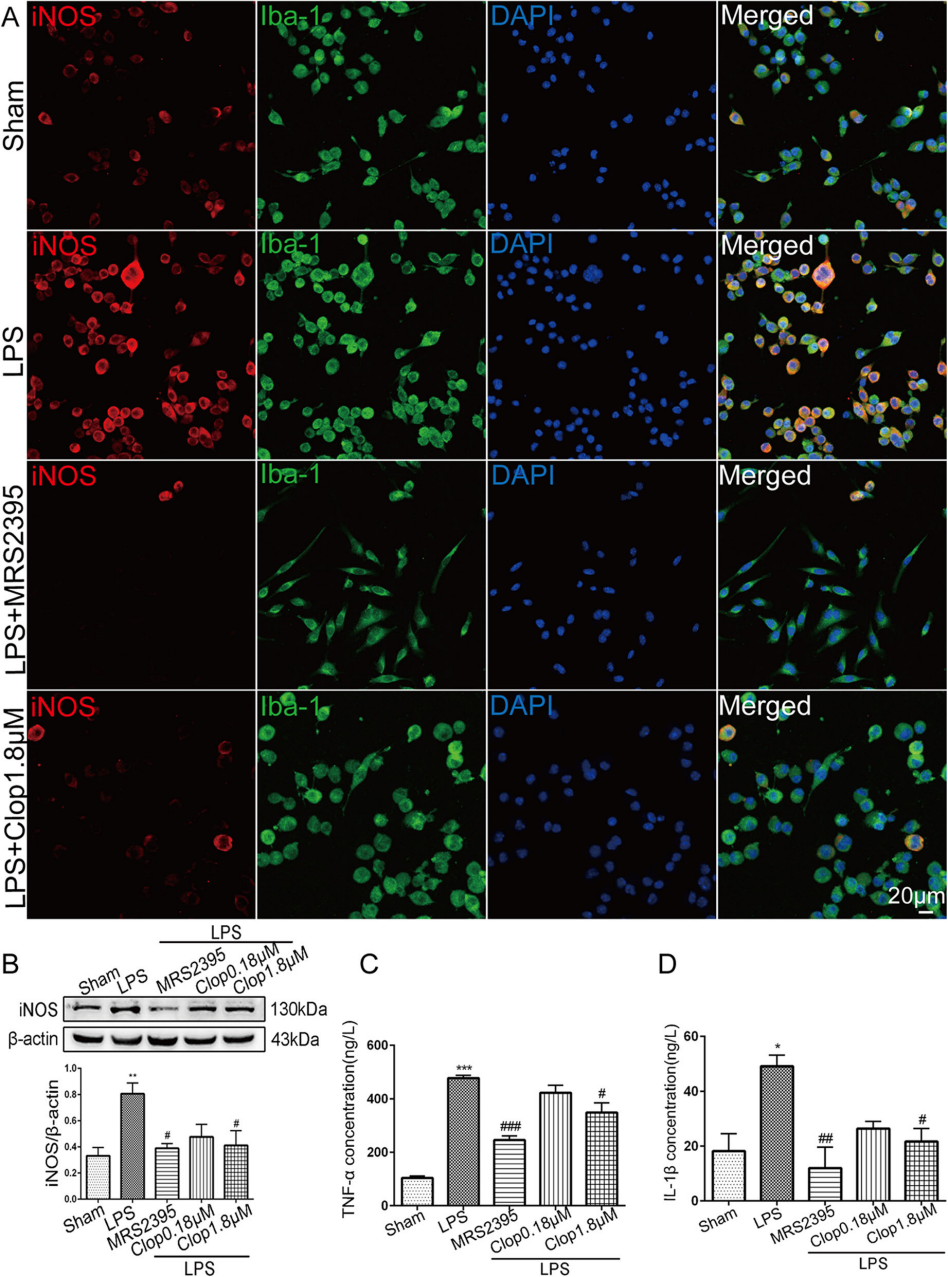
Effect of P2Y12R on LPS-stimulated BV-2 microglial morphological changes and inflammatory cytokine production. **a** Double immunofluorescence staining images of iNOS with Iba-1 in BV-2 cells stimulated with LPS and pretreated with MRS2395/clopidogrel (1.8 μM). Scale bar: 20 μm. **b** Representative Western blot showing iNOS expression in LPS-stimulated BV-2 cells pretreated with the P2Y12 antagonists MRS2395 (20 μM) and clopidogrel (0.18 and 1.8 μM). The band intensity is presented relative to that of β-actin. Data are presented as mean ± SEM of three independent experiments; **p < 0.01 compared with sham group; ^#^*p* < 0.05 compared with the LPS group. **c**, **d** ELISA of IL-1β and TNF-α release from LPS-stimulated BV-2 cells pretreated with MRS2395 and clopidogrel. Data are presented as mean ± SEM of three independent experiments; *p < 0.05 and ***p < 0.001 compared with the sham group; ^#^*p* < 0.05, ^##^*p* < 0.01, and ^###^*p* < 0.001 compared with the LPS group

BV-2 cells were preincubated with the P2Y12R inhibitors MRS2395 (20 μM) and clopidogrel (0.18 and 1.8 μM) for 2 h followed by LPS stimulation for 24 h. BV-2 cells in the sham group most frequently showed an ovoid shape; LPS treatment (1 μg/ml) induced an activated state exhibiting a hypertrophic phenotype; and MRS2395 pre-treatment significantly prevented this cellular transformation, with these BV-2 cells exhibiting elongated cell bodies (Fig. 9a). However, pre-treatment with clopidogrel (1.8 μM) had almost no effect on cell morphological changes (Fig. 9a). The Western blot and immunofluorescence data revealed that MRS2395 and clopidogrel (1.8 μM) pre-treatment significantly reduced the protein expression of iNOS (*p* < 0.05, LPS + MRS2395, 0.39 ± 0.035; *p* < 0.05, LPS ± Clop1.8 μM, 0.41 ± 0.111 vs. LPS, 0.81 ± 0.083) in LPS-treated BV-2 cells (Fig. 9a, b). ELISA was performed to quantify the release of IL-1β and TNF-α in the medium. Both MRS2395 and clopidogrel (1.8 μM) significantly reduced the amount of IL-1β (*p* < 0.01, LPS + MRS2395, 11.99 ± 7.638 ng/l; *p* < 0.05, LPS ± Clop1.8 μM, 21.61 ± 4.751 ng/l vs. LPS, 49.01 ± 4.138 ng/l) and TNF-α (*p* < 0.001, LPS + MRS2395, 245.31 ± 16.165 ng/l; *p* < 0.05, LPS ± Clop1.8 μM, 348.13 ± 37.312 ng/l vs. LPS, 477.74 ± 9.952 ng/l) released into the medium (Fig. 9c, d). However, incubation with 0.18 μM clopidogrel had no significant effect on the production of iNOS, IL-1β, or TNF-α.

## Discussion

We found the following new results in the present study. First, using an animal model of chronic NTG treatment-induced CM, we provided the first evidence that microglial P2Y12R in the TNC participates in the pathogenesis of CM. Then, we showed that microglial P2Y12R regulated activated microglial morphological changes and inflammatory factor production in the TNC via the RhoA/ROCK pathway during CM. Finally, we presented the first evidence that the RhoA/ROCK2 pathway contributes to the development of CM.

Recurrent NTG administration is known to induce acute and chronic hypersensitivity [28, 40], mimicking the clinical characteristics of CM patients, in whom tactile allodynia may occur both between and during migraine attacks. NTG treatment might also cause other features of migraine, such as photophobia, facial grimace behaviors, and changes in meningeal blood flow in mice [41] and the upregulation of calcitonin gene-related peptide (CGRP) in the TNC, dura mater, and blood [39]. Thus, systemic recurrent NTG administration can be used to establish a reliable animal model of CM. There are sex differences in migraine, which are mainly due to the role of estrogen [42, 43]. Studies show that estrogen fluctuations affect transmitters of pain signaling such as gamma aminobutyric acid and serotonin [44], and mediate neuronal activation in the TNC [45]. Previous reports have proposed that the NTG-induced CM model can be successfully established in both male and female mice. To avoid the influence of estrogen, we selected male mice for further study. In addition, previous literature shows no sex effect on P2Y12R activation [22].

Studies in animal models of neuropathic, inflammatory, and cancer pain have shown that both the mRNA and protein levels of spinal microglial P2Y12R are markedly increased [21, 22, 29, 46, 47]. We confirmed the upregulation of P2Y12R in the TNC in our NTG-induced CM model. Since the changes in P2Y12R were consistent with the upregulation of microglia in the TNC, we predicted that the changes in P2Y12R in the TNC in our CM model may be due to the activation of microglia. We found that the location of P2Y12R was highly restricted to microglia in the TNC. Regarding the expression and cellular localization of P2Y12R in the trigeminocervical complex, a previous study [20] reported that P2Y12R expression was increased in rats with tongue cancer pain and that P2Y12R was expressed on both microglia and neurons in the Vc. These differences might be due to the different animals and pain models used in studies. In addition, P2Y12R has also been found in the trigeminal ganglion (TG) [48–50]. We did not directly investigate the role of P2Y12R in the TG in our present study; however, since we systemically administered P2Y12R antagonists, we cannot rule out the potential effect on the TG. Therefore, this potential role of peripheral P2Y12R in CM needs to be further explored.

Upregulation of microglial P2Y12R is known to promote the development of neuropathic or inflammatory pain. Our study first confirmed the association between P2Y12R and CM. Our behavioral data indicated that blocking P2Y12R activation by repeated intraperitoneal administration of its selective inhibitors MRS2395 and clopidogrel leads to the attenuation of NTG-induced basal tactile allodynia; however, acute injection of antagonists did not relieve pain behaviors significantly. The analgesic effect of MRS2395 and clopidogrel has been determined in neuropathic and inflammatory pain models [19].

Clopidogrel is a well-known antithrombotic thienopyridine prodrug, whose active metabolites irreversibly inhibit P2Y12R activity in platelets. Clinically, clopidogrel administration requires several consecutive days to achieve a therapeutic effect because the active metabolites of clopidogrel need to be converted in the liver [51]. Therefore, we administered clopidogrel for five consecutive days so that functional P2Y12R inhibition would be effective. The slow onset of analgesic effects of clopidogrel may be attributed to the limitation of the active metabolites transfer from plasma to the trigeminovascular system. Previous studies suggest that intraperitoneal administration of 20 mg/kg clopidogrel in mice induces a clinically relevant prolongation of platelet aggregation and bleeding time [31], and that a 10 mg/kg intraperitoneal injection achieved a satisfactory effect on relieving inflammatory pain in rats [19]. Consistent with these results, we found that intraperitoneal injection of 15 mg/kg clopidogrel results in the most significant relief of allodynia in mice with CM. Therefore, upregulation of microglial P2Y12R in the TNC may be critical for the development of CM.

We systemically administered P2Y12R inhibitors and both the microglia and platelets should be considered the targets of the inhibitors. The metabolites of clopidogrel were observed to cross the blood-brain barrier [29]. We observed that micrcoglial P2Y12R protein levels in the TNC were inhibited by using their antagonist systemically (Additional file 1: Figure S1). A previous study has demonstrated that the effect of clopidogrel on microglial function in the CNS is unlikely contributed to the suppression of platelets activity, because non-P2Y12R-dependent platelet antagonists, acetylsalicylic acid and heparin, did not reduce the function of microglia [31]. Our finding and the previous result support microglia as a main site of action in the TNC. However, it would take a long way for clopidogrel to target microglial P2Y12R in the TNC, thus the inhibitory effects of the drugs on central sensitization may still contribute to their action in the periphery (i.e., platelet) or the CNS vasculature and their indirect reduction of microglial P2Y12R expression. Platelets activation may be involved in triggering migraine attacks through 5-HT metabolism, NO, and a number of pro-inflammatory cytokines, including interleukins 1, 6, and 8 (IL-1, IL-6, IL-8) and TNF-α [52, 53]. Therefore, the effect of P2Y12R inhibitors on platelets activation may also be the driver of pain-associated behaviors, and this potential role needs to be further explored.

The precise details of how microglial P2Y12R promotes CM remain to be determined. CGRP, a key neuropeptide in the trigeminal system, is a critical contributing factor to peripheral and central sensitization [39], which is involved in CM. C-fos, an immediate early gene that has been widely used as a marker of neuronal activity, is also involved in central sensitization [54]. We observed that the levels of CGRP and c-fos were increased in the TNC in association with severe basal hypersensitivity in our CM model, and that both P2Y12R antagonists, MRS2395 and clopidogrel, suppressed the expression of CGRP and c-fos. Since CGRP and c-fos are produced by neurons, we predict that the inhibitory effect of microglial P2Y12R on CGRP and c-fos may be due to involvement in microglial-neuronal interactions. Studies have shown that microglia can interact with neurons through chemotaxis, in which microglia may extend the tips of their branched processes to neighboring sensory neurons [29], and produce various pro-inflammatory cytokines. Therefore, we focused on the relationship between P2Y12R and microglia. Previous research found that P2Y12R can regulate microglial morphological changes and inflammation [18, 22, 34]. In this study, we confirmed that recurrent NTG injection induced microglial morphological alterations and that iNOS production in the TNC was partially suppressed by P2Y12R antagonists. These findings suggest that NTG stimulates microglial activation leading to cytoskeletal rearrangement through P2Y12R. In addition, p38 MAPK, downstream of P2Y12R, appears to be involve in the expression of various pro-inflammatory factors such as IL-1β, IL-6, and TNF-a as well as iNOS [55]. We previously found that p38 MAPK was related to CM [10]; thus, the regulatory effect of P2Y12R on microglial inflammation in the TNC may be dependent on p38 MAPK.

In addition, clopidogrel decreased microglial process retraction and iNOS production in the TNC in CM mice via inhibiting microglial P2Y12R activity, which may be a mechanism of relieving migraine attacks. We also confirmed the effect of P2Y12R on microglial activation in vitro. Both MRS2395 and clopidogrel suppressed the production of inflammatory factors, including IL-1β, TNF-α, and iNOS, in LPS-stimulated BV-2 microglia. A previous study reported that clopidogrel may reduce the levels of serotonin or pro-inflammatory cytokines such as IL-1, 6, 8, and TNF-α released by platelets [56]. Therefore, the effect of clopidogrel on inflammation in the peripheral and central nervous systems through inhibiting P2Y12R may contribute to the attenuation of CM.

In the in vitro study, we found that MRS2395 but not clopidogrel significantly changed cell morphology. Inhibition of P2Y12R on platelets requires the conversion of clopidogrel to active metabolites, but studies in endothelial cells showed that either thienopyridine metabolites or thienopyridines themselves reduced cell proliferation and the production of vasoactive NO and PGI 2 [57–59]. We did not analyze the mechanism of how clopidogrel itself react with BV-2 cells in the study. Knowing that there are other crucial purinergic receptors such as P2X4R and P2X7R presented in a high expression on microglial surface, so we hypothesized that the effect exerted by clopidogrel on BV-2 cells may be contributed to all purinergic receptors presented on BV-2 cells or is a result of the stimulation of other unknown targets. The explanation of how clopidogrel itself reacts with BV-2 cells is complicated and requires further insightful analysis.

Studies have examined whether the activation of RhoA/ROCK could be regulated by P2Y12R [18]. In the present study, we confirmed that the RhoA/ROCK pathway acted downstream of P2Y12R in the CM model. Data have shown that ROCK activity may be associated with the mechanism of neuropathic, inflammatory, and cancer pain [38, 60, 61]. A recent study showed that the activation of ROCK participated in cortical spreading depression (CSD) [62], which is a critical pathology of migraine. Therefore, it is reasonable to consider that ROCK plays a role in migraine. In our study, we found that the levels of GTP-RhoA and ROCK2 in the TNC were increased after repeated NTG injection and that this upregulation promoted the maintenance of CM, as demonstrated by the use of the ROCK2 inhibitor. Fasudil, a nonselective antagonist of ROCK2 that has a greater potency toward ROCK2 than toward other kinases [63], attenuated hyperalgesia in CM mice and suppressed the expression of CGRP in the TNC by inhibiting the activation of the RhoA/ROCK pathway. Consistent with the effect of P2Y12R in the TNC, RhoA/ROCK may also be involved in microglial-neuronal crosstalk. ROCK appears to regulate neuronal activity by affecting the production of nitric oxide [38], and plays a critical role in microglial activation via p38 MAPK [18]. We examined whether inhibition of ROCK2 activity suppressed the changes in activated microglial morphology and inflammation in the TNC. ROCK2 activity is known to be involved in multiple events, including neuritogenesis, synaptogenesis, axon guidance, axon elongation, and the retraction cycle. These functions partly contribute to the rearrangement of actin and thus to cellular motility and cytoskeletal structure [26]. Thus, ROCK2 mediates microglial morphological alterations and may also contribute to cytoskeletal rearrangement. In addition, RhoA/ROCK can regulate the activation of p38 MAPK [18]; thus, the inhibitory effect on iNOS production in the TNC in the CM model may depend on the regulation of p38 MAPK activation. Based on these results, we confirmed that microglial P2Y12R regulates microglial activation through the RhoA/ROCK pathway in the TNC during CM. In addition, P2Y12R may also have an impact on the activity of sensory neurons in the TNC, but how it participates in microglial-neuronal crosstalk in the TNC remains ambiguous. Future work needs to be performed to confirm the role of P2Y12R in this crosstalk.

## Conclusion

In conclusion, we demonstrated the roles of microglial P2Y12R in the TNC in CM. These roles include affecting microglial morphological changes and inflammatory factor production in the TNC through the RhoA/ROCK pathway and suppressing the expression of CGRP and c-fos in the TNC. These results suggest a new mechanism of CM and help identify that P2Y12R antagonists, such as clopidogrel, might be a new therapeutic strategy for treating CM in the clinic.

## Supplementary information

**Additional file 1:** **Figure S1.** Representative immunofluorescence images of P2Y12R in the TNC shows clopidogrel treatment inhibited the upregulation of P2Y12R following NTG administration.

## Data Availability

The datasets used and analyzed during the current study are available from the corresponding author on reasonable request.

## Abbreviations

ANOVA: Analysis of variance
CGRP: Calcitonin gene-related peptide
CM: Chronic migraine
CNS: Central nervous system
CSD: Cortical spreading depression
ELISA: Enzyme-linked immunosorbent assay
IL-1β: Interleukin-1β
iNOS: Inducible nitric oxide synthase
IR: Immunoreactive
LPS: Lipopolysaccharide
NTG: Nitroglycerin
ROCK: Rho-associated coiled-coil-containing protein kinase
TNC: Trigeminal nucleus caudalis
TNF-α: Tumor necrosis factor-α
