# Supplementary material for: P2Y12 receptor mediates microglial activation via RhoA/ROCK pathway in the trigeminal nucleus caudalis in a mouse model of chronic migraine
Source: J Neuroinflammation. 2019 Nov 13;16:217. doi: 10.1186/s12974-019-1603-4 (PMC6854723; doi:10.1186/s12974-019-1603-4)
Supplement: Supplementary file 1 — Additional file 1: Figure S1. Representative immunofluorescence images of P2Y12R in the TNC shows clopidogrel treatment inhibited the upregulation of P2Y12R following NTG administration. [file 12974_2019_1603_MOESM1_ESM.pdf]

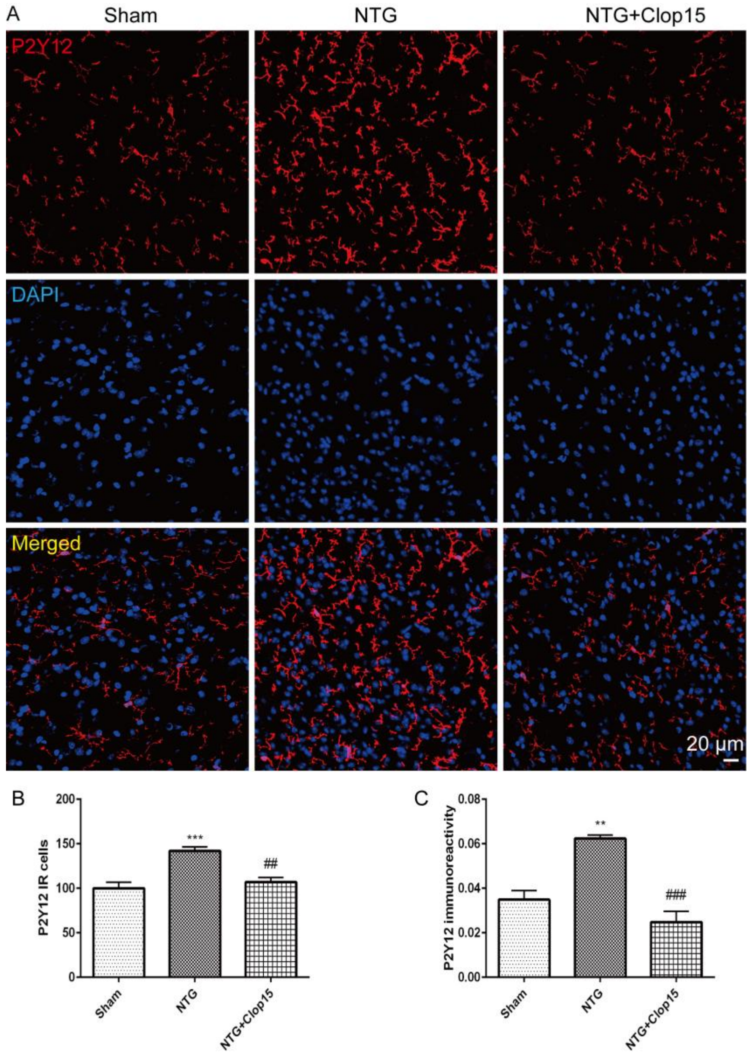

Fig. S1. Upregulation of P2Y12R in the NTG group in the TNC was inhibited by clopidogrel administration. a Representative immunofluorescence images of P2Y12R in the TNC shows changes of P2Y12R after NTG and clopidogrel administration. **b-c** Quantitative analysis of the number of P2Y12R immunoreactive (ir) cells (**b**) and P2Y12R immunoreactivity (**c**) in the TNC. Both the number of P2Y12R ir cells and P2Y12R immunoreactivity in the NTG group were increased compared with the sham group. Clopidogrel (15 mg/kg) treatment significantly reversed the

upregulation of P2Y12R in the TNC. Data are represented as mean ± SEM, n=6 per group, **$p<0.01$ and ***$p<0.001$ compared with sham group, $^{\#\#}p<0.01$ and $^{\#\#\#}p<0.001$ compared with NTG group. Scale bar: 20 μm.
